# Supplementary material for: Aging in mice alters regionally enriched striatal astrocytes
Source: Nat Commun. 2025 Sep 26;16:8496. doi: 10.1038/s41467-025-63429-8 (PMC12475473; doi:10.1038/s41467-025-63429-8)
Supplement: Supplementary file 1 — Supplementary information [file 41467_2025_63429_MOESM1_ESM.docx]

**Aging in mice alters regionally enriched striatal astrocytes**

^1^Kay E. Linker*, ^3^Violeta Duran-Laforet, ^1^Matthias Ollivier, ^1^Xinzhu Yu^Ψ^, ^3^Dorothy P. Schafer and ^1,2^Baljit S. Khakh

^1^Department of Physiology and ^2^Department of Neurobiology David Geffen School of Medicine, University of California Los Angeles, Los Angeles USA CA 90095-1751. ^3^Department of Neurobiology, Brudnick Neuropsychiatric Research Institute, University of Massachusetts Chan Medical School, Worcester, MA, 01605, USA.

# **Supplementary figures**

# **13 Supplementary figures with legends (in the pages that follow)**

#
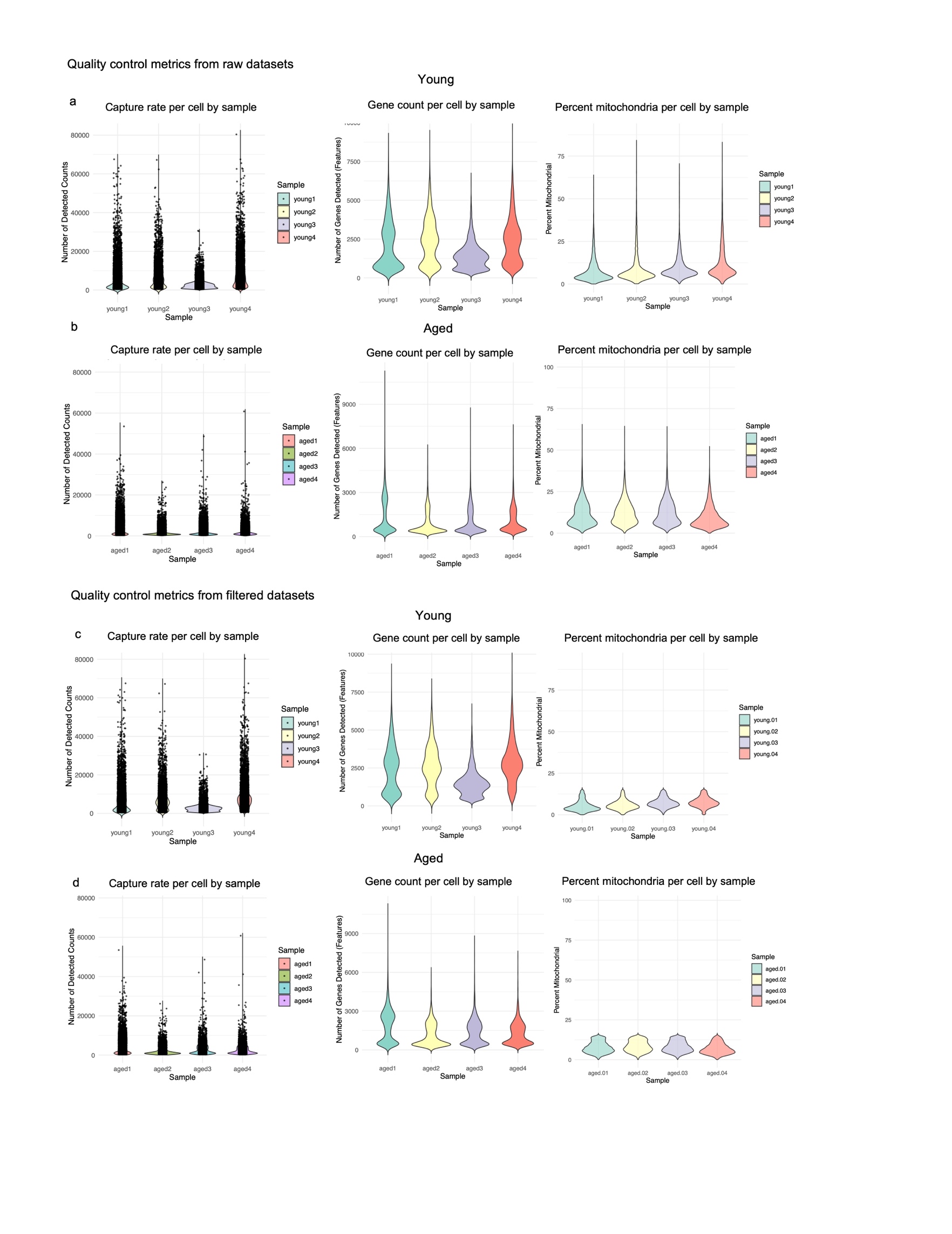


**Supplementary Fig 1:** **Data quality control metrics across aged and young samples before and after filtering steps.** Panels (**a**) and (**b**) show quality control metrics across young (**a**) and aged (**b**) in raw, pre filtered data. **a.** The capture rate (the number of detected UMI counts per cell) is depicted across four young (2-months-old) samples, followed by the number of unique genes per young sample. The third plot in the panel shows the percent mitochondrial genes of total genes (genes with mt-xx/number of unique genes per cell *100) per young sample from the raw data sets. **b.** The capture rate is depicted across four aged (18-months-old) samples, followed by the number of unique genes per aged samples. The final plot in the panel shows the percent mitochondrial genes (genes with mt-xx/number of unique genes per cell *100) per young sample per cell from the raw data sets. Panels (**c**) and (**d**) show quality control metrics across young (**c**) and aged (**d**) from filtered data (see methods). **c.** The capture rate (the number of detected UMI counts per cell) is depicted across four young samples, followed by the number of unique genes per young samples. The third plot in the panel shows the percent mitochondrial genes of total genes (genes with mt-xx/number of unique genes per cell *100) for each young sample from the filtered data sets. **d.** The capture rate is depicted across four aged (18-months-old) samples, followed by the number of unique genes per aged samples. The third plot in the panel shows the percent mitochondrial genes of total genes per aged sample from the filtered data sets. Aged samples have lower amounts of genes per cell and higher mitochondrial genes per cell and this has been previously shown in the literature of past aging scRNAseq papers^1,2^.

**
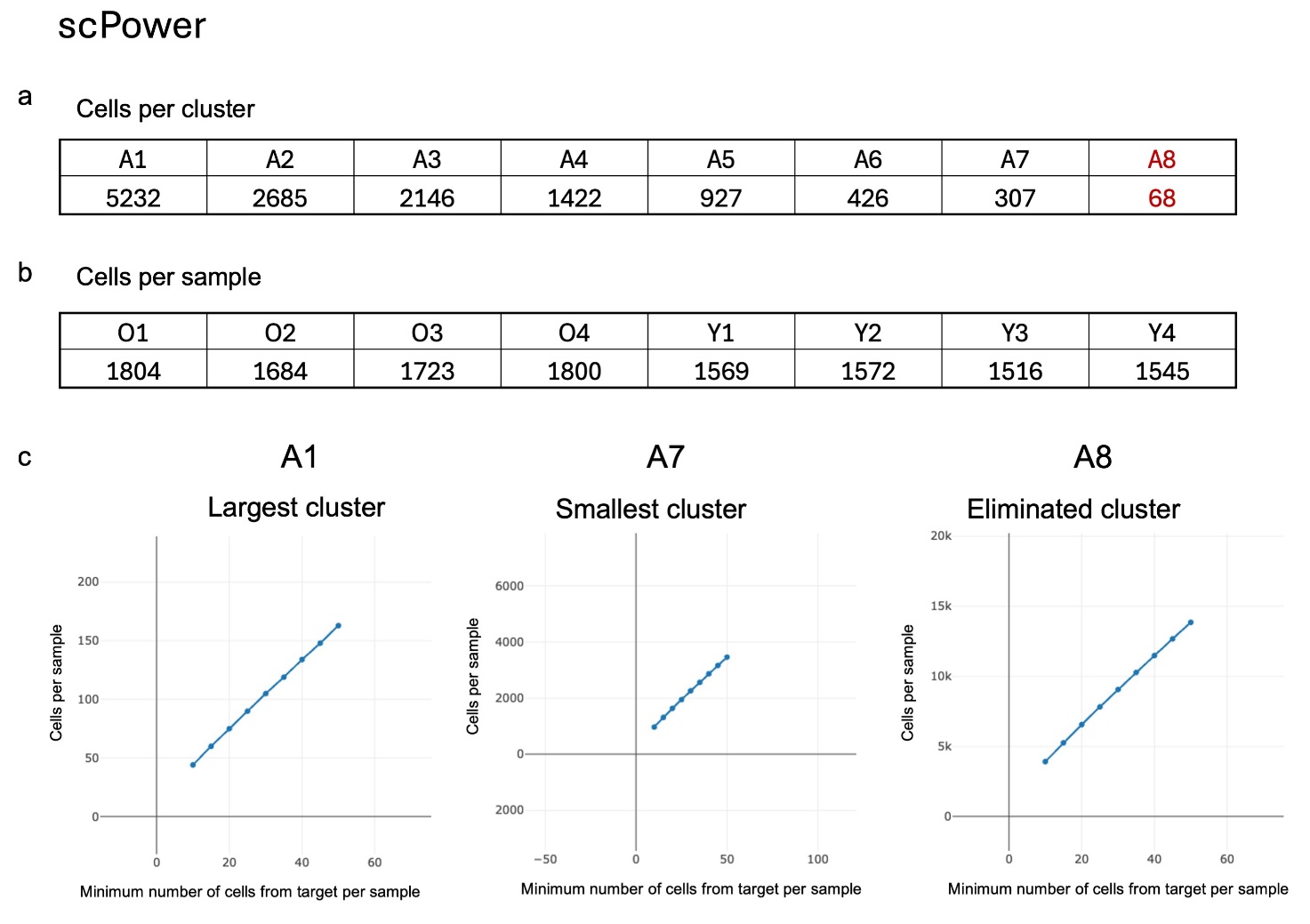
**

**Supplementary Fig 2:** **We conducted** **power analysis using the scPower algorithm to assess the capability of our dataset to identify astrocyte subtypes in the striatum**. **a.** The number of cells per cluster from the largest cluster (A1) to the smallest clusters (A7 and A8). We removed A8 from the dataset because we did not have enough power to identify this cell cluster. **b.** The total number of cells per sample, categorized by age and condition (*e.g.,* young *vs* old). **c.** We used scPower to identify the number of cells needed per sample and per subset to have enough statistical power to identify the individual subset. ScPower uses a multinomial model and models the distribution of cell-type proportions and tests whether there is a significant difference in the distribution of cell types between conditions^3^. The y axis is the total number of cells needed per sample, the x axis is the minimum cells needed per subtype, and the line indicates the acceptable range. The power analysis indicates that while some clusters (*e.g.,* A8) are underpowered due to low cell counts, the overall dataset still maintains sufficient statistical power to detect and analyze most astrocyte subtypes under study. The eliminated cluster (A8) was identified as having too few cells for robust statistical analysis and was therefore excluded from downstream analyses throughout the work.

#
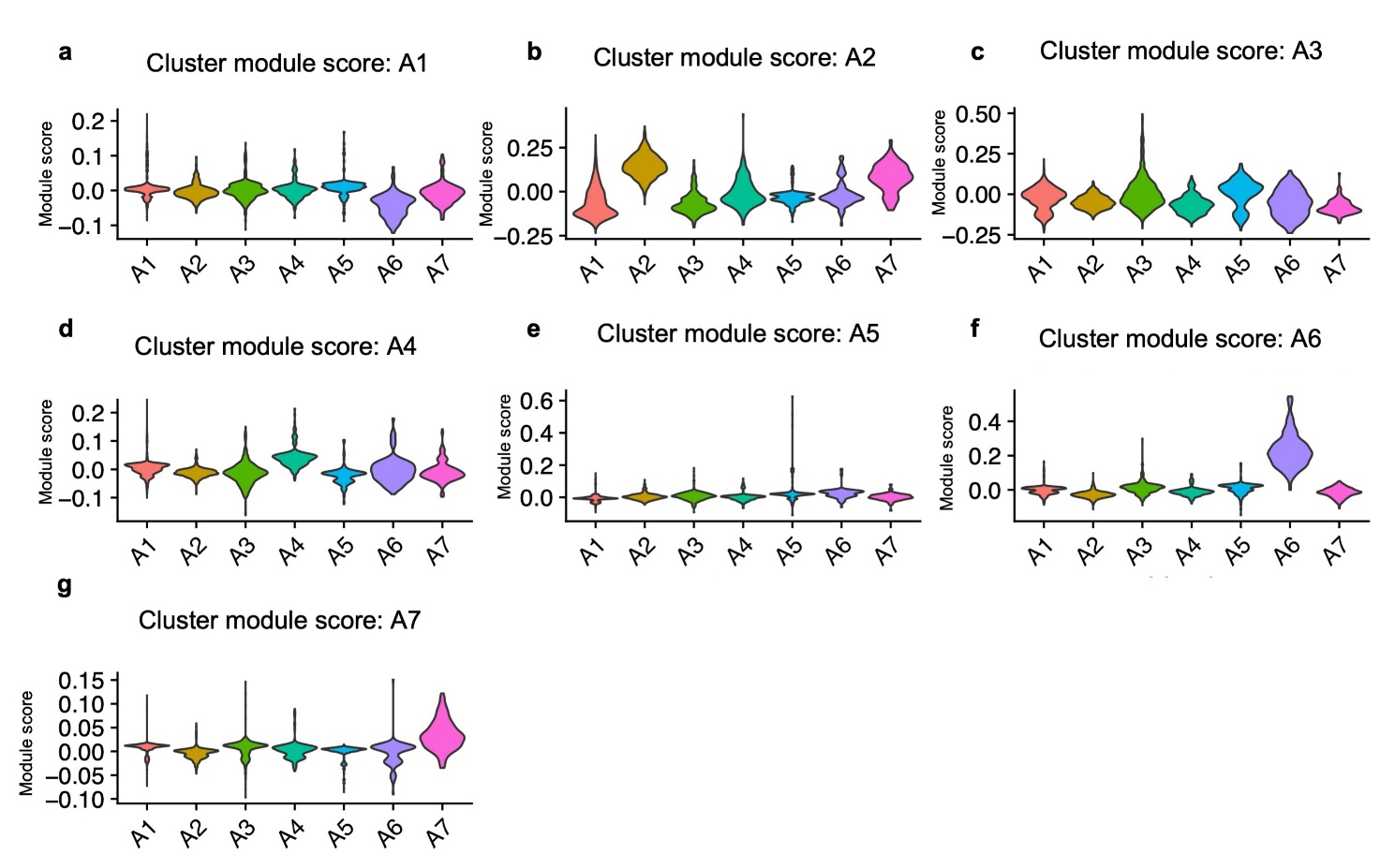


**Supplementary Fig 3:**  **To** **visually represent cluster integration and molecular distinction with the single-cell RNA sequencing and MERFISH data sets we calculated cluster module scores for each astrocyte subtype.** Cluster score was calculated from the top 10 DEGs using Seurat’s module score calculation. Seurat selects a set of control genes to serve as a baseline, these are selected based on the distribution of their expression level, to match the distribution of the dataset. The number of control genes was set to 100, to ensure statistical robustness, and are randomly selected in bins based on their average expression level. To calculate the module score, Seurat subtracts the average module expression from the control expression, per individual cell. This shows a pattern of gene expression across the data sets. Panels a-g, the cluster score was calculated the same way. (**a**) Top 10 cluster defining genes for subset A1 (as a module score as described above) are shown in a violin plot across astrocyte subsets. (**b**) Top 10 cluster defining genes for subset A2 (as a module score as described above) are shown in a violin plot across astrocyte subsets. (**c**) Top 10 cluster defining genes for subset A3 are shown in a violin plot across astrocyte subsets. (**d**) Top 10 cluster defining genes for subset A4 are shown in a violin plot across astrocyte subsets. (**e**) Top 10 cluster defining genes for subset A5 are shown in a violin plot across astrocyte subsets. (**f**) Top 10 cluster defining genes for subset A6 are shown in a violin plot across astrocyte subsets. (**g**) Top 10 cluster defining genes for subset A7 are shown in a violin plot across astrocyte subsets.

#
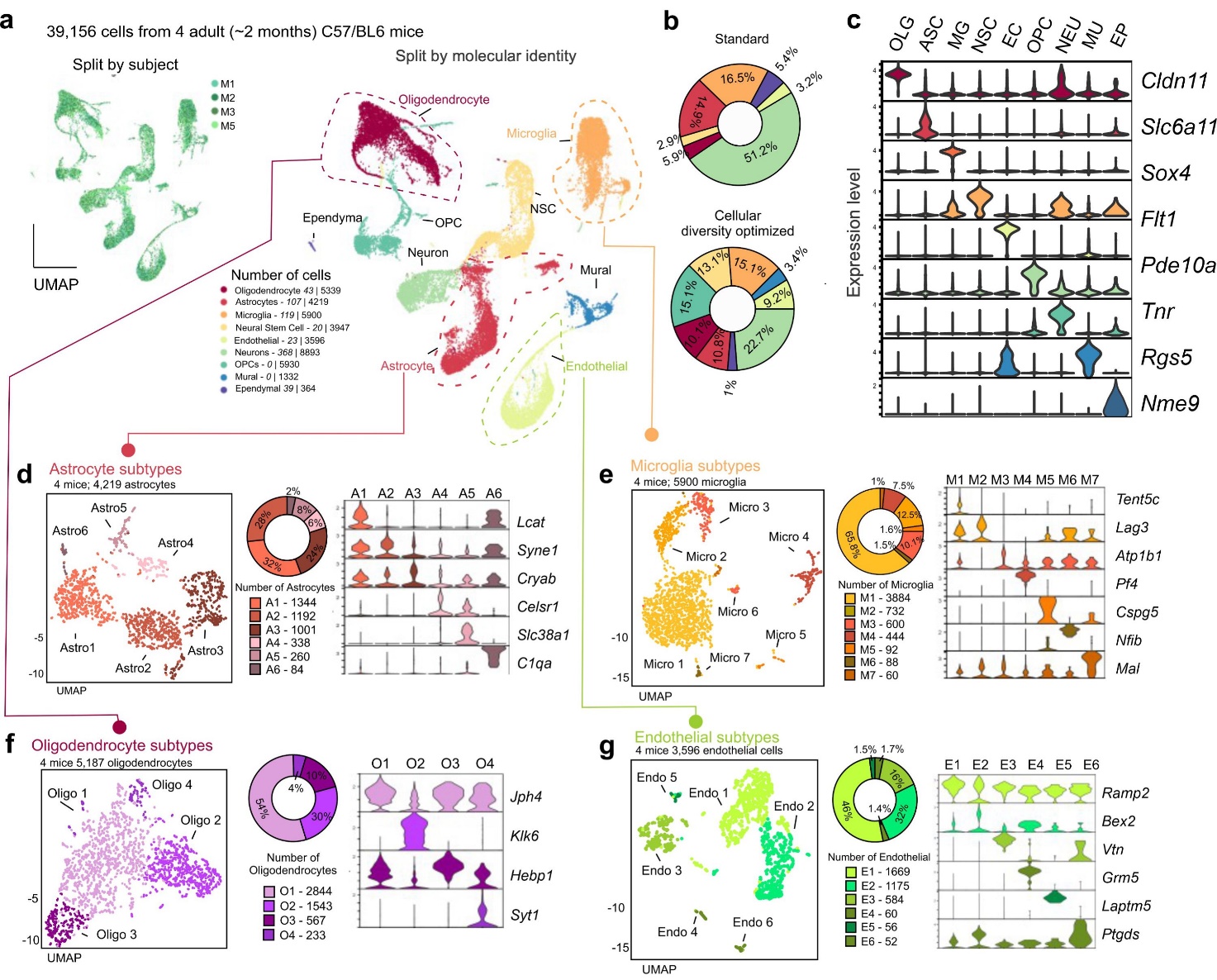


**Supplementary Fig 4: Single-cell analysis of young (2-month-old) striatal cells. a.** Single-cell RNA sequencing (scRNAseq) was used to evaluate transcriptomes of striatal cells from young mice (n = 4 mice). The plots show Uniform Manifold Approximation and Projection (UMAP) split by age and molecular identity. **b.** Cell percentages in the standard scRNAseq sample preparation^4^, versus our cellular diversity optimized protocol using a 20 μm pore filter. **c.** Known cell markers identified cell classes, and a violin plot of select markers is shown (OLG = oligodendrocyte, ASC = astrocyte, MG = microglia, NSC = neural stem cell, EC = endothelial cell, OPC = oligodendrocyte precursor cell, NEU = neuron, MU = mural cell, EP = ependyma cell). The astrocyte (**d**), microglia (**e**), oligodendrocyte (**f**) and endothelial (**g**) cell classes were individually subset from the broader data set and a second tier of community Louvain detection was performed for individual cell type analysis. There are 6 young astrocytes subtypes (**d**), 7 young microglia subtypes (**e**), 6 young endothelial subtypes (**g**) and 4 young oligodendrocytes subtypes (**f**). Individual subtypes contribute varying percentages to the total cell class, and this quantification is indicated for each with a pie chart. Under each pie chart is the number of cells in each subcluster, and the pie chart indicates the percentage each subcluster contributes to the total. Each sub-cluster had identifying markers, which are represented in the violin plots to the right of each pie chart.

#
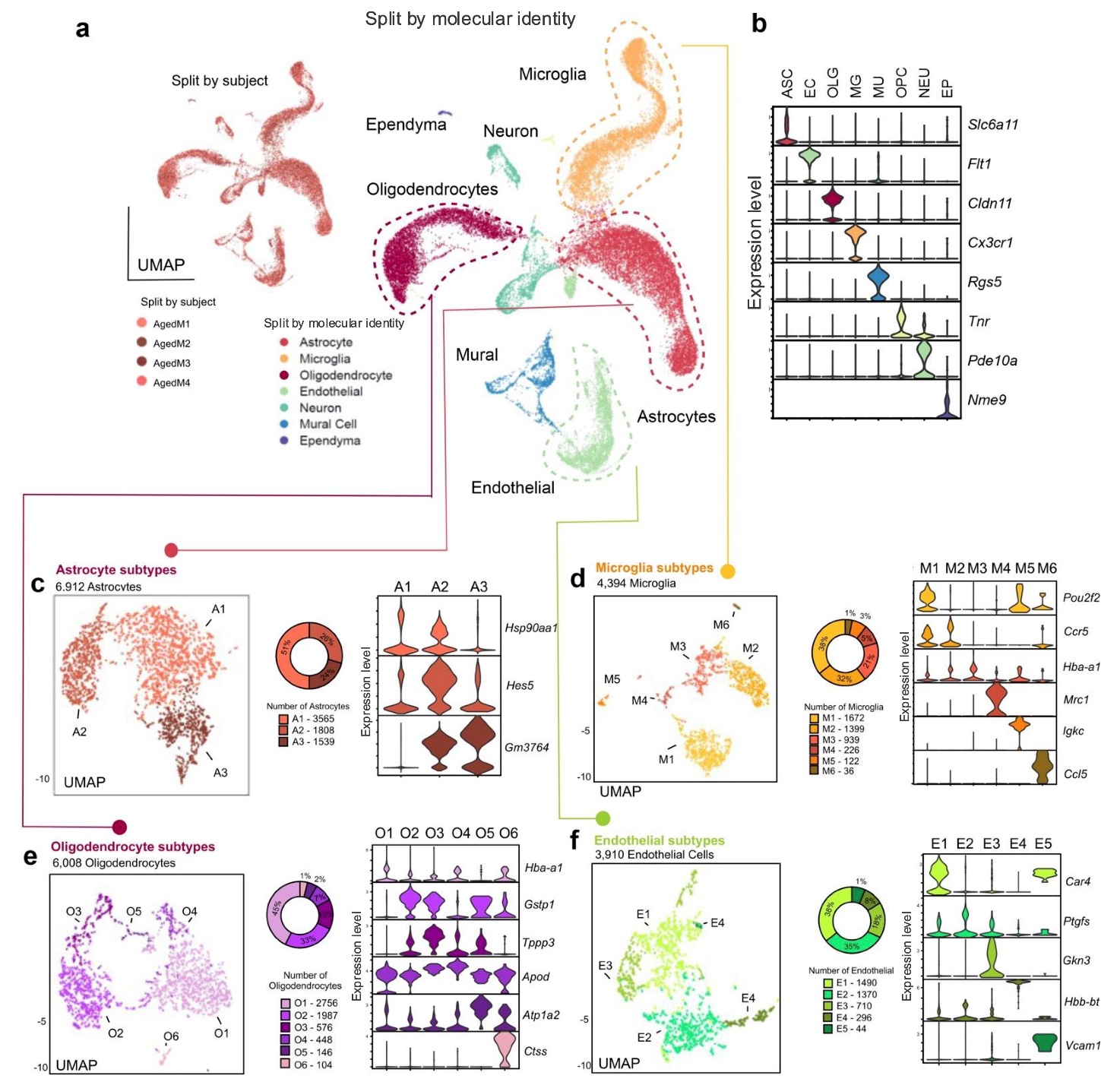


**Supplementary Fig 5: Single-cell analysis of aged striatal cells alone.** Single-cell RNA sequencing (scRNAseq) was used to evaluate transcriptomes of aged striatal cells (n = 4 mice). Uniform Manifold Approximation and Projection (UMAP) split by age (**a**) and molecular identity. Known cell markers identified cell classes, and a violin plot of select markers is shown (OLG = oligodendrocyte, ASC = astrocyte, MG = microglia, NSC = neural stem cell, EC = endothelial cell, OPC = oligodendrocyte precursor cell, NEU = neuron, MU = mural cell, EP = ependyma cell). (**b**). The astrocyte (**c**), microglia (**d**), oligodendrocyte (**e**) and endothelial (**f**) cell classes were individually subset from the broader data set and a second tier of community Louvain detection for higher granularity. There are 3 aged astrocytes subtypes (**c**), 6 aged microglia subtypes (**d**), 5 aged endothelial subtypes (**f**) and 6 aged oligodendrocytes subtypes (**e**). Individual subtypes contribute varying percentages to the total cell class, and this quantification is indicated in each associate pie chart. Under each pie chart is the number of cells in each subcluster, and the pie chart indicates the percentage each subcluster contributes to the total. Each sub-cluster had identifying markers, which are represented in the violin plots to the right of each pie chart.

#
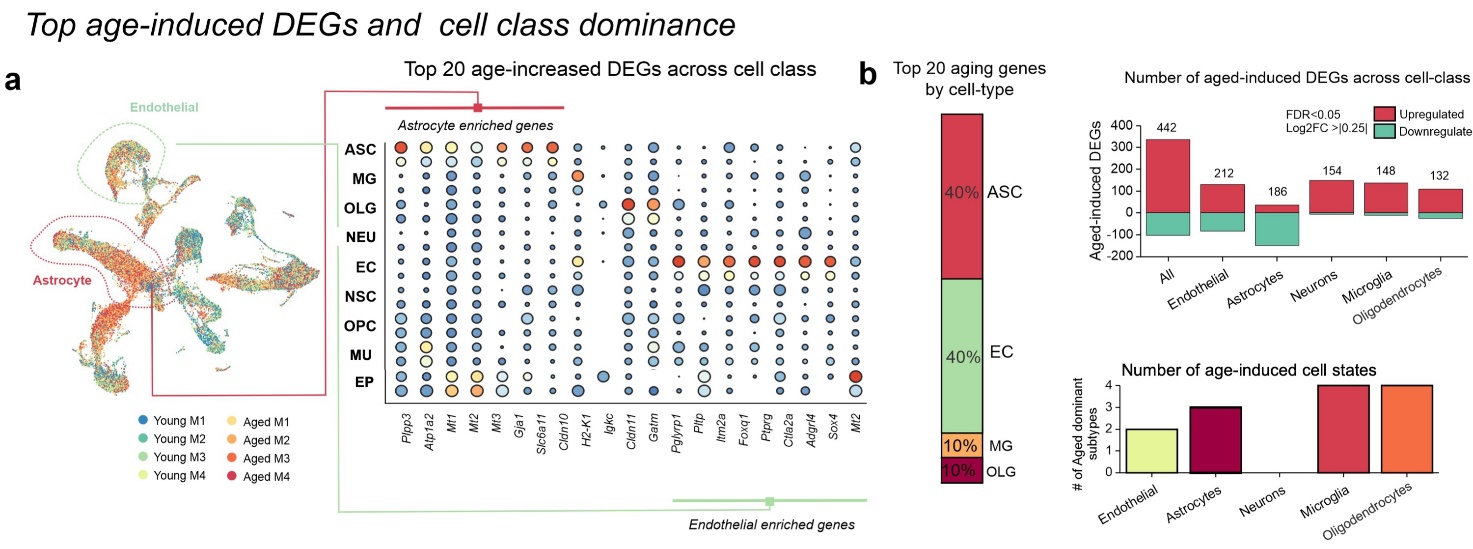


**Supplementary Fig 6: Top age-induced DEGs within the striatum irrespective of cell class and their expression across all cell-types**. **a.** UMAP of all cells split by age (n = 4 young mice, n = 4 aged mice). Accompanied by dot plot showing top 20 age induced differentially expressed genes, and their expression across each subtype (OLG = oligodendrocyte, ASC = astrocyte, MG = microglia, NSC = neural stem cell, EC = endothelial cell, OPC = oligodendrocyte precursor cell, NEU = neuron, MU = mural cell, EP = ependyma cell). **b.** 80% of differentially expressed aging genes were enriched in either astrocytes or endothelial cells Endothelial cells and astrocytes had the most DEGs across cell types, and new age-induced cell states. These cell types data that had newly clustered subsets that were aged dominant subtypes (Figure 1, Supplementary Figure 8). This is the number of new aged-dominant subtypes in the merged datasets, demonstrating significant cell shifts that were molecularly distinct enough to Louvain cluster into a new molecular subset.

**
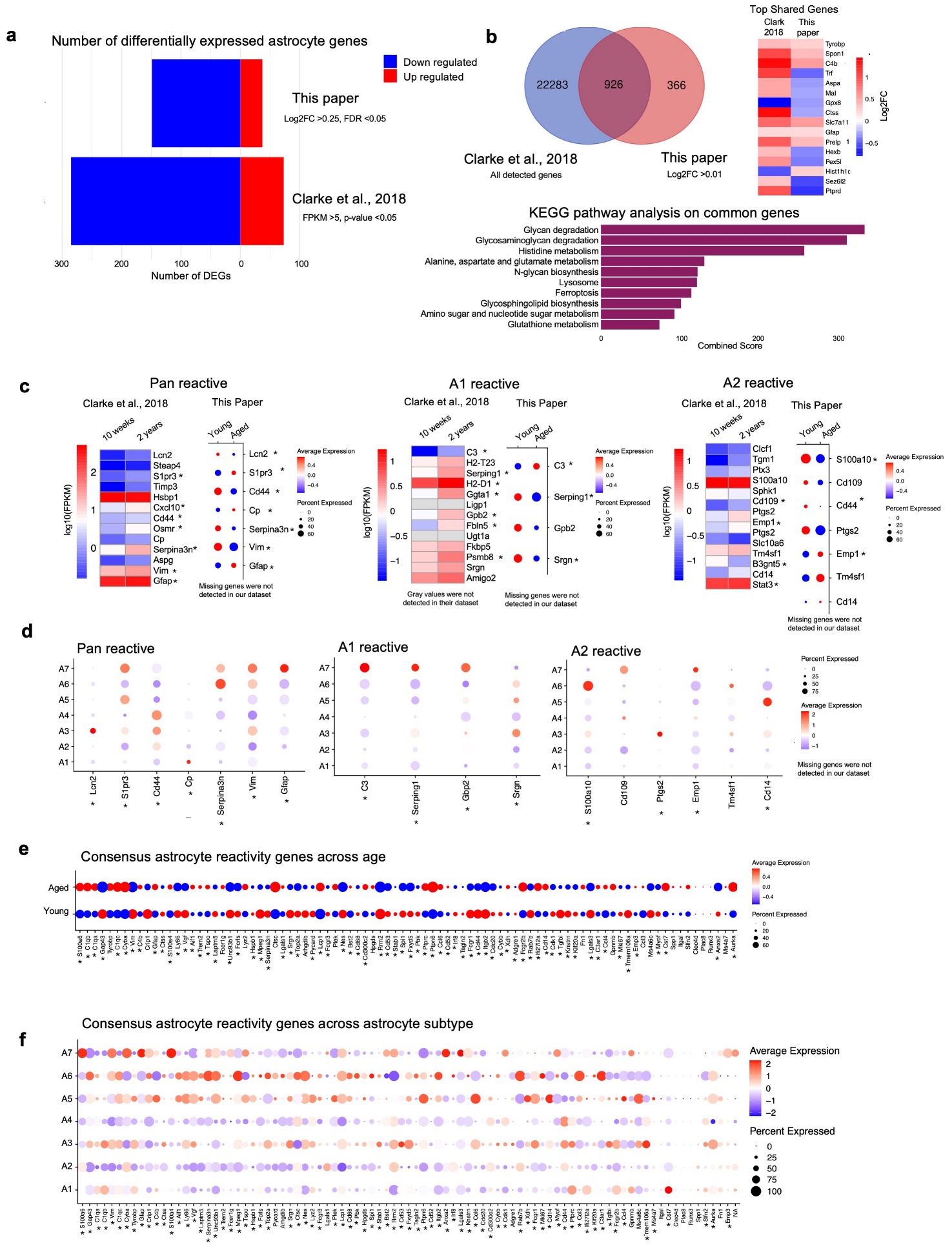
**

**Supplementary Fig 7: Comparison of differentially expressed genes between our data sets and past work.** (**a**) Comparison of differentially expressed genes in our paper (cut off = Log2FC >0.25, FDR <0.01), and Clarke et al., 2018 (cut off = FPMK >5, FDR <0.05). (**b**) The venn diagram shows all detected genes from the Clarke et al., paper (blue) and our paper (red) There were 926 common genes. The heatmap shows the common 16 genes from our paper with the cut off Log2FC >0.25 and FDR <0.05, and the Clarke paper with the cut off FPKM >5, and FDR <0.05. This heatmap show Log2FC in the Clarke et al paper^5^ (2 years compared to 10 weeks) and our paper (18 months compared to 2 months). We used Enrichr to perform KEGG pathway analysis on the top 16 common genes. (**c**) We compared the expression Pan reactive, A1 reactive and A2 reactive expression in Clarke paper and our paper using heatmaps (visualizes the Log10(FPKM) from the Clarke paper) and dotplots show the average expression of the markers in this paper between old and young. Genes that are missing from normalized counts in our dataset due to low expression were not included. Asterix indicates significant differential expression using MAST DEG analysis (FDR <0.05). (**d**) We compared Pan reactive, A1, and A2 markers across the different astrocyte subsets. Genes that are missing from normalized counts in our dataset due to low expression were not included. Asterix indicates significant differential expression across astrocyte subtypes using Wilcoxon rank sum analysis (Seurat Find all markers, FDR <0.05). (**e**) We compared the 170 genes in the consensus astrocyte reactivity genes (cARGs)^6^ across age. 88 genes were detected and using MAST DEG differential expression analysis 87% of these genes were significantly differentially expressed (* indicates FDR < 0.05). (**f**). We compared the 170 genes in the consensus astrocyte reactivity across astrocyte subtype. 88 genes were detected and using Wilcoxon rank sum analysis (Seurat Find all markers) 91% of these genes were significantly differentially expressed (* indicates FDR <0.05).

#
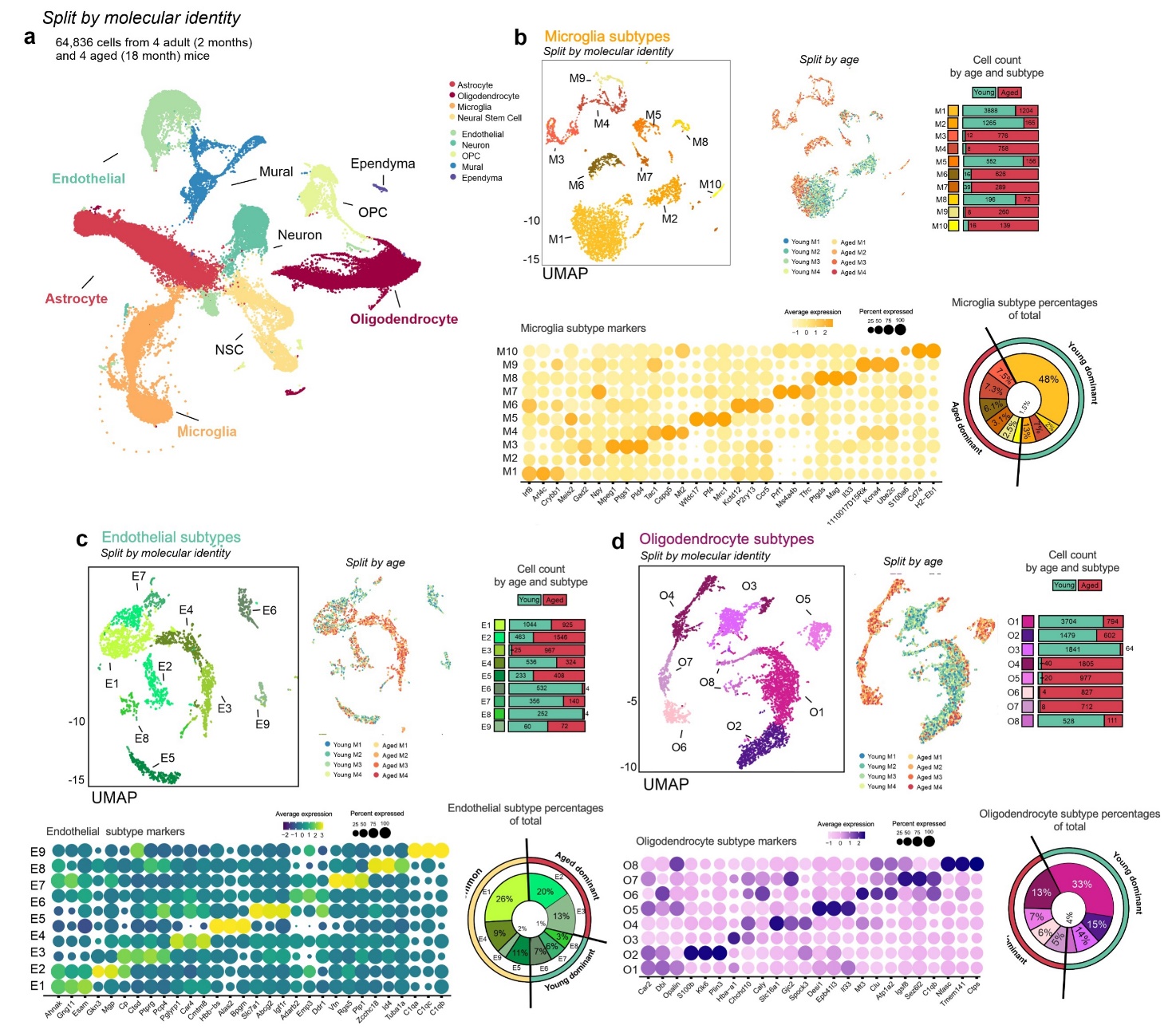


**Supplementary Fig 8: Single-cell RNAseq analysis of other major glial populations and endothelial cells in the striatum with age. a.** UMAP of all cell clusters within the striatum split by molecular identity (n = 4 young mice, 4 aged mice). UMAP of microglia (**b**), endothelial (**c**) or oligodendrocytes (**d**) shows putative subtypes identified with Louvain clustering (resolution = 0.1), split by molecular identity and age. The numbers of each cell type derived from young (green) or old (red) for each glial subtype (M1-10; E1-9; O1-6) is shown to display contribution of young or aged cells to each astrocyte subcluster. The relative percentage of each glia subcluster commits to the total amount of each respective glia (inner pie) and if the subcluster is dominated by cells from aged, young or neither (common) samples (outer pie). Subclusters were determined to be young or aged dominant if >70% of the cells within that cluster came from young or aged mice. Expression of three distinct subtype markers for each subcluster are shown, with the scaled average expression level indicated by circle color, and the percentage of cells within a cluster expressing the marker indicated by circle size.

**
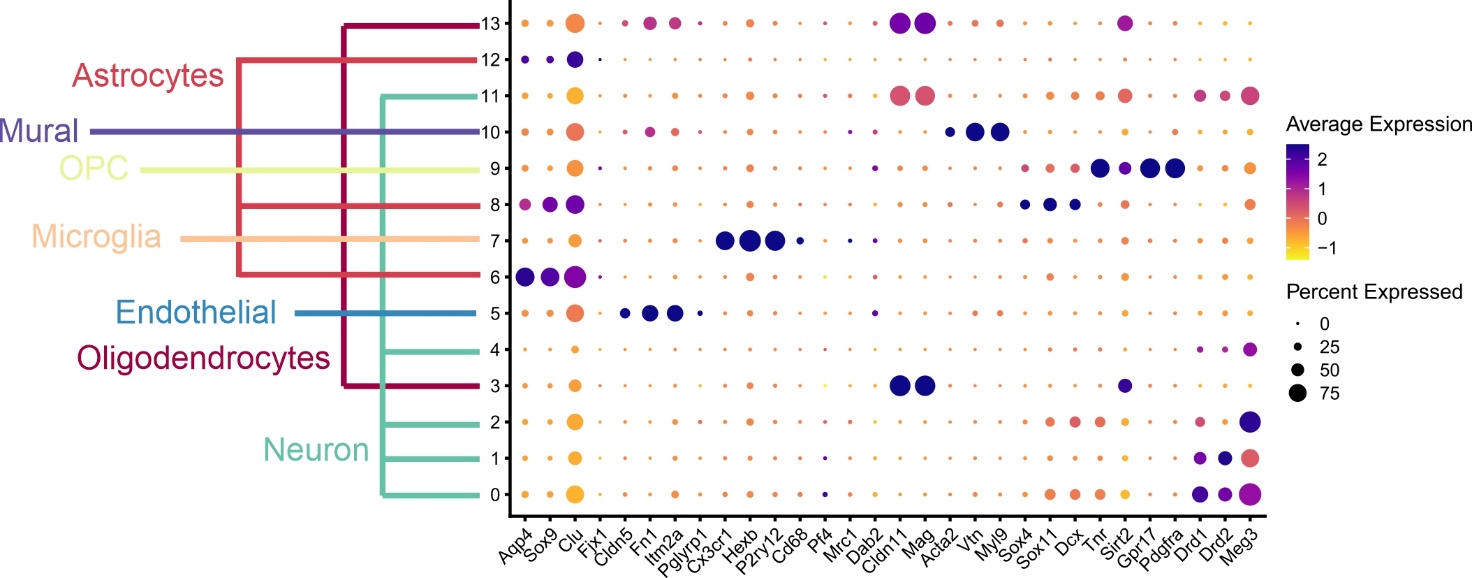
**

**Supplementary Fig 9: MERFISH cell type identification through established markers.** Canonical markers for individual cell classes were used to identify MERFISH cell populations. Each cell class marker expression is shown across all cell populations using a dot plot, where the color of each dot indicates the scaled average expression, and the size of the dot indicates the percentage of cells in the population expressing the marker.

#
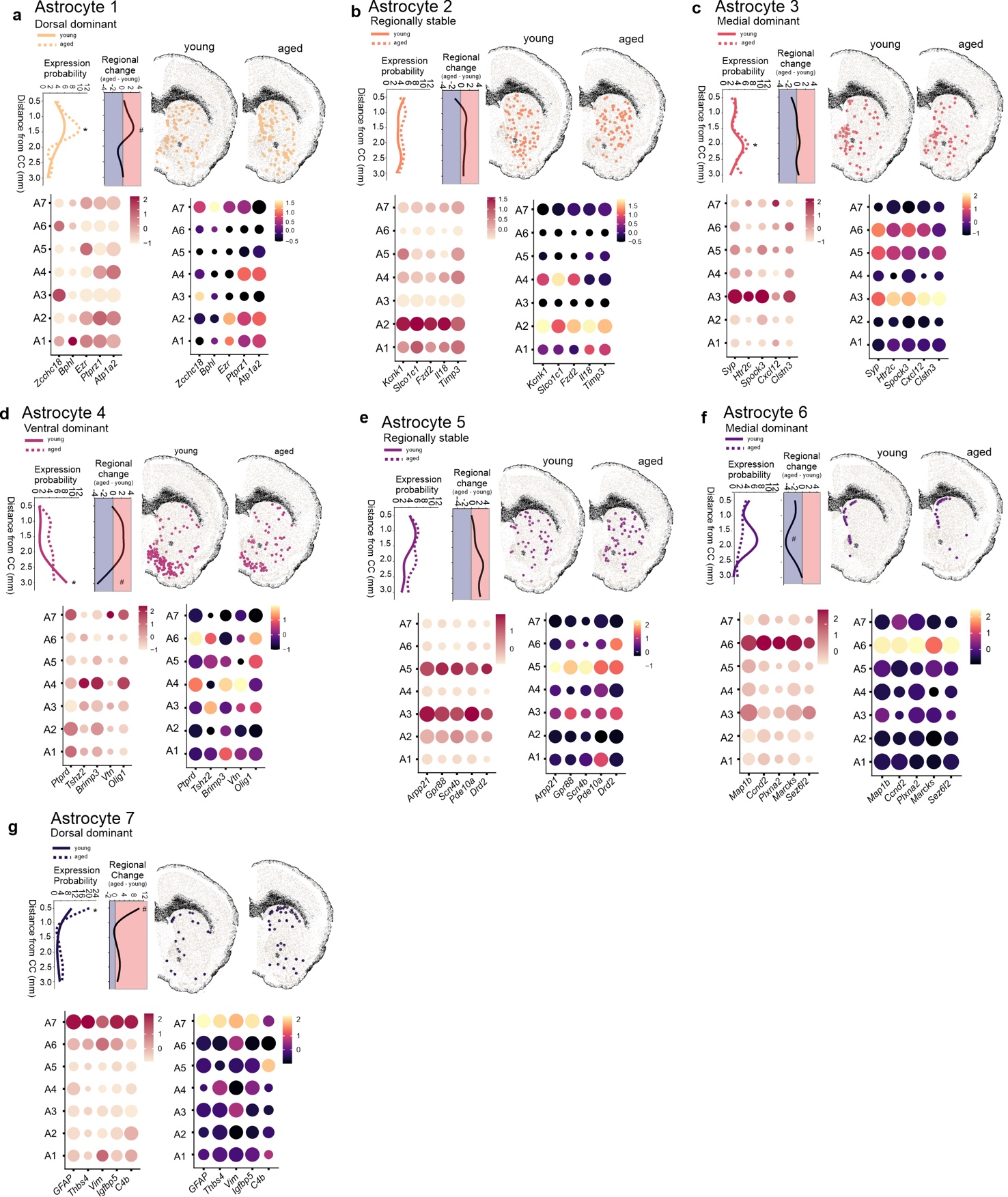


**Supplementary Fig 10: MERFISH astrocyte subtype markers and spatial allocations. a-g.** Each astrocyte subtype shows the astrocyte subtype expression probability and the change in this probability with age (n = 4 mice). This is followed by representative images of XY spatial expression of individual astrocyte subtypes across the striatum. Under the spatial representations of each astrocyte subtype, two dot plots of astrocyte subtype marker expression are shown. The first dot plot shows the marker expression in MERFISH astrocytes, and the second dot plot shows marker expression in scRNAseq astrocytes. **a.** Astrocyte subtype 1 has higher population density in the dorsal striatum. **b.** Astrocyte subtype 2 is a regionally stable population. **c.** Astrocyte subtype 3 is medial dominant. **d.** Astrocyte subtype 4 is ventral dominant (**a-d**). Five individual markers for A1-A4 expression patterns across astrocyte subtypes are visualized in dot plots in both scRNAseq and MERFISH. **e.** Astrocyte subtype 5 is a regionally stable population. **f.** Astrocyte subtype 6 is a population that is medial dominant and resides near ventricle borders in young mice. **g.** Astrocyte subtype 7 is dorsal dominant in young mice and increases in population in aged mice. **a-g.** Five individual markers for A1-A7 expression pattern across astrocyte subtypes are visualized in dot plots in both scRNAseq and MERFISH.


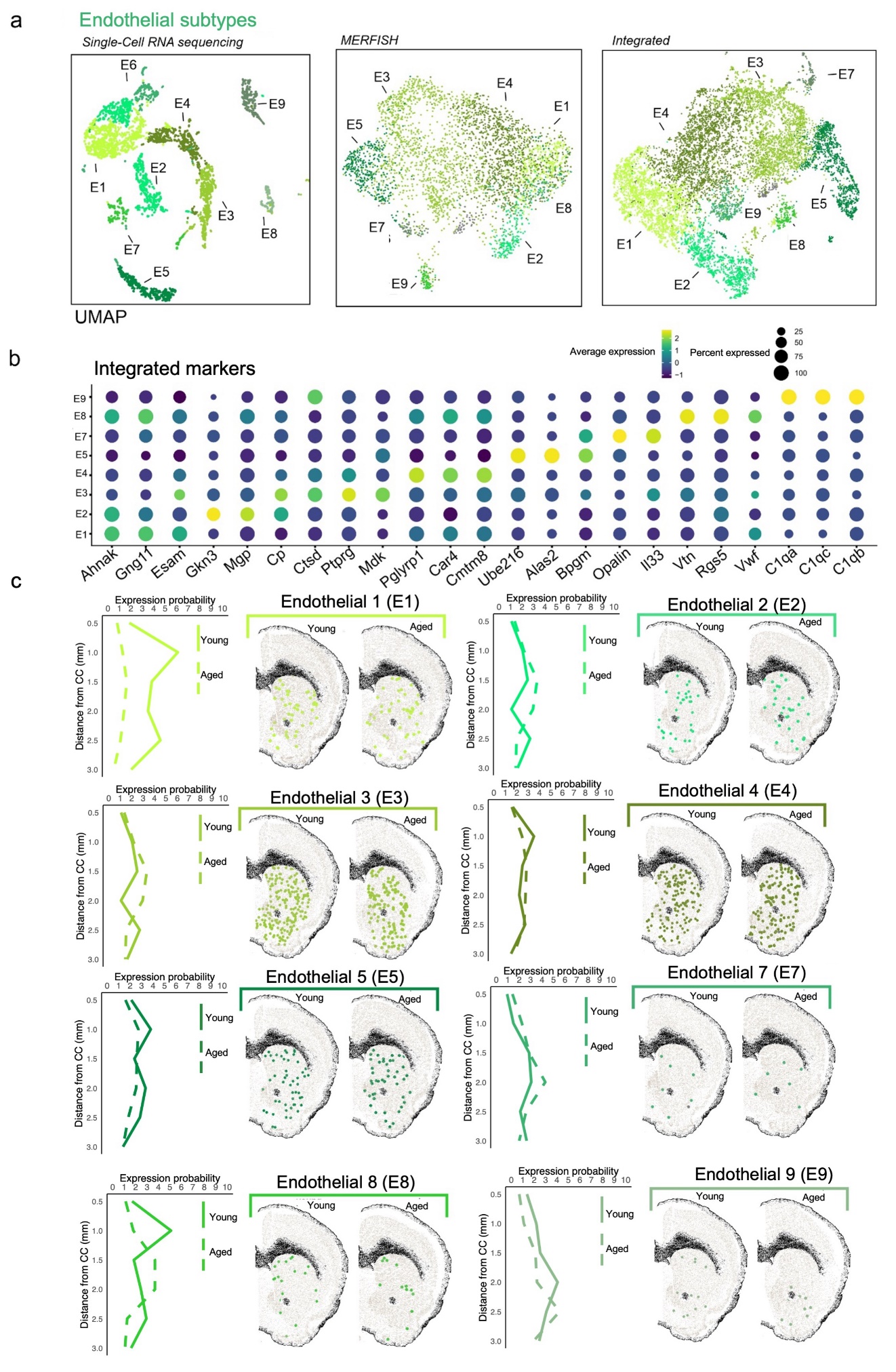


**Supplementary Fig 11: MERFISH endothelial subtype markers and spatial allocations. a.** UMAPs of striatal endothelial subtypes in scRNAseq, MERFISH and integrated data sets from young (n = 4 mice) and aged mice (n = 4 mice). Each embedding used Louvain clustering to derive endothelial subclusters, after subsetting from larger striatal cell datasets. Endothelial subset 6 was not identified in the MERFISH dataset **b.** Dot plot shows three markers for each individual endothelial subtype and their expression; circle size indicates the percentage of cells that express the marker and circle color indicates the scaled average expression. **c.**  Endothelial expression probability was calculated for each subset identified in the MERFISH data set. This endothelial expression probability quantification was calculated by the number of endothelial within a subcluster (E1-5, E7-E9) within each 500 μm subregion (0-5) (X_E1...E9_ within Y_0...5_) divided by the total number of endothelial cells within that 500 μm section (Σ_total_) normalized to the total number of endothelial cells within each respective subcluster (σ_E1...E9_) ([(X_E1,..E8_ within Y_0...5_/Σ_total_)/σ_E1...E9_ ]). The regional change is quantified by subtracting the young endothelial expression probability by the aged expression probability. The solid line represents young endothelial expression probability, and the dashed line represents the aged subtype expression probability. Representative spatial map of individual endothelial subtypes (E1-5, E7-9) across the striatum.


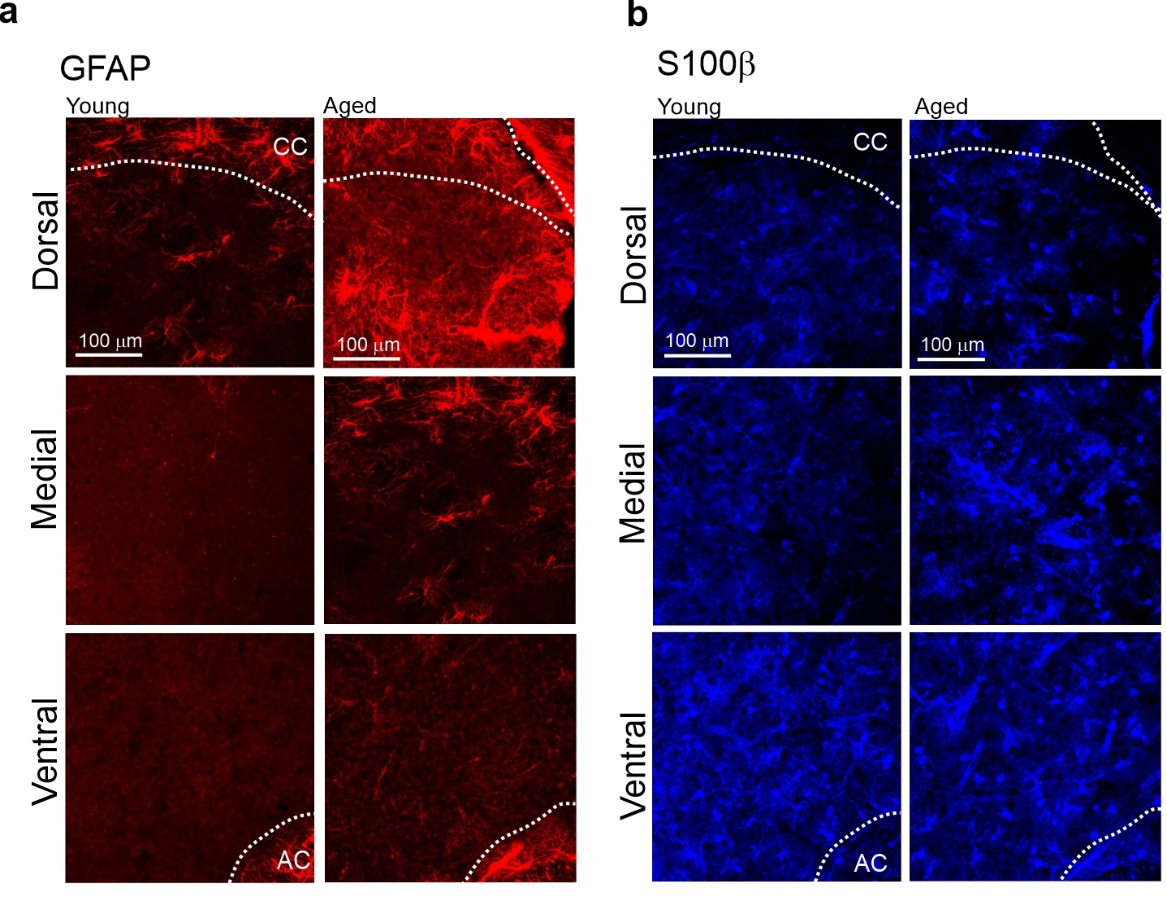


**Supplementary Fig 12. IHC for GFAP and S100β across the striatum in young and aged mice. a.** Representative images of the dorsal, medial and ventral striatum are shown for young and aged mice. GFAP expression coverage in image areas of 500 μm^2^ in the dorsal, medial, and ventral striatum for young (2 month) and aged (18 months) mice (n = 4-5 mice). **b.** Representative images of S100β expressing cells in image areas of 500 μm^2^ in the dorsal, medial, and ventral striatum for young (2 month) and aged (18 months) mice (n = 4-5 mice).

#
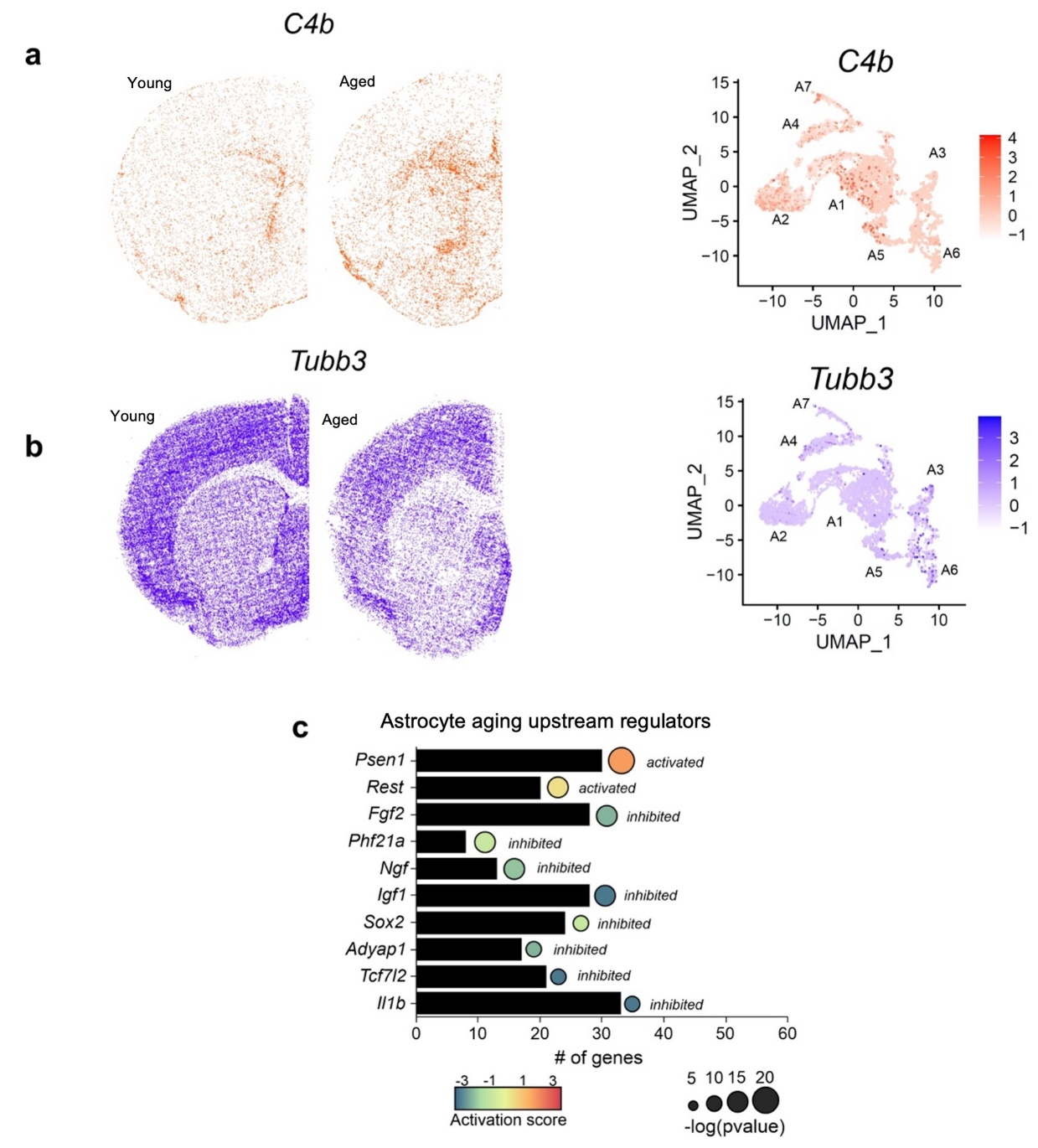


**Supplementary Fig 13. Additional molecular properties of astrocytes from scRNAseq and MERFISH data.** **a, b.** Representative images of MERFISH RNA counts shows age increases *C4b* in the dorsal striatum and decreases Tubb3 in the ventral striatum. Single-cell UMAP feature plot shows *C4b* or *Tubb3* expression across each subcluster. *C4b* goes up with age and demarcates age dominant astrocytes (A1, A5); *Tubb3* decreases with age and demarcates young dominant (A6, A3). **c.** IPA upstream regulator analysis was performed on age-induced DEGs in striatal astrocytes from the scRNA sequencing dataset. Each black bar indicates the number of genes per pathway or upstream regulator, circle size indicates the -log(pvalue), and color within the circle indicates the activation score number for each upstream regulator.

# **References cited in the Supplementary figures**

1 Allen, W. E., Blosser, T. R., Sullivan, Z. A., Dulac, C. & Zhuang, X. Molecular and spatial signatures of mouse brain aging at single-cell resolution. *Cell* **186**, 194-208.e118, doi:10.1016/j.cell.2022.12.010 (2023).

2 Ximerakis, M. *et al.* Single-cell transcriptomic profiling of the aging mouse brain. *Nature neuroscience* **22**, 1696-1708, doi:10.1038/s41593-019-0491-3 (2019).

3 Schmid, K. T. *et al.* scPower accelerates and optimizes the design of multi-sample single cell transcriptomic studies. *Nature communications* **12**, 6625, doi:10.1038/s41467-021-26779-7 (2021).

4 Wu, Y. E., Pan, L., Zuo, Y., Li, X. & Hong, W. Detecting Activated Cell Populations Using Single-Cell RNA-Seq. *Neuron* **96**, 313-329 (2017).

5 Clarke, L. E. *et al.* Normal aging induces A1-like astrocyte reactivity. *Proceedings of the National Academy of Sciences of the United States of America* **115**, E1896-E1905 (2018).

6 O'Shea, T. M. *et al.* Lesion environments direct transplanted neural progenitors towards a wound repair astroglial phenotype in mice. *Nature communications* **13**, 5702, doi:10.1038/s41467-022-33382-x (2022).
