# Supplementary material for: Aging in mice alters regionally enriched striatal astrocytes
Source: Nat Commun. 2025 Sep 26;16:8496. doi: 10.1038/s41467-025-63429-8 (PMC12475473; doi:10.1038/s41467-025-63429-8)
Supplement: Supplementary file 2 — Description of Additional Supplementary Files [file 41467_2025_63429_MOESM2_ESM.docx]

**Description of Additional Supplementary Files**

**Supplementary data 1:** Genes used in the MERFISH panel.

**Supplementary data 2:** Contribution of each sample to each individual cell type in single cell and MERFISH, and a comparison of the subcluster percentages in single cell and MERFISH for astrocyte and endothelial cells.

**Supplementary data 3:** Comparison of genes detected in published striatal astrocyte RiboTag RNAseq data (Chai et al., 2017) and the genes detected in this study for astrocytes. The file also shows the results of IPA pathway analysis and all cluster defining markers for astrocyte subsets from this study.

**Supplementary data 4:** Full CellChat table for astrocytes, microglia and endothelial cells with p-values, log2Fc, and probability scores.

**Supplementary data 5:** Full gene lists used in human comparisons as part of Figure 4h and astrocyte DEGs from scRNAseq summarized in Fig. 4a. Age-related DEGs for oligodendrocytes, microglia and endothelial cells are also reported.
