# Supplementary material for: Aging in mice alters regionally enriched striatal astrocytes
Source: Nat Commun. 2025 Sep 26;16:8496. doi: 10.1038/s41467-025-63429-8 (PMC12475473; doi:10.1038/s41467-025-63429-8)
Supplement: Supplementary file 8 — Reporting Summary [file 41467_2025_63429_MOESM8_ESM.pdf]

nature portfolio

Corresponding author(s): Kay E. Linker, Baljit S. Khakh

Last updated by author(s): Jul 18, 2025

Reporting Summary

Nature Portfolio wishes to improve the reproducibility of the work that we publish. This form provides structure for consistency and transparency in reporting. For further information on Nature Portfolio policies, see our Editorial Policies and the Editorial Policy Checklist.

Statistics

For all statistical analyses, confirm that the following items are present in the figure legend, table legend, main text, or Methods section.

n/a	Confirmed	
☐	☒	The exact sample size (*n*) for each experimental group/condition, given as a discrete number and unit of measurement
☐	☒	A statement on whether measurements were taken from distinct samples or whether the same sample was measured repeatedly
☐	☒	The statistical test(s) used AND whether they are one- or two-sided<br>*Only common tests should be described solely by name; describe more complex techniques in the Methods section.*
☐	☒	A description of all covariates tested
☐	☒	A description of any assumptions or corrections, such as tests of normality and adjustment for multiple comparisons
☐	☒	A full description of the statistical parameters including central tendency (e.g. means) or other basic estimates (e.g. regression coefficient) AND variation (e.g. standard deviation) or associated estimates of uncertainty (e.g. confidence intervals)
☐	☒	For null hypothesis testing, the test statistic (e.g. *F*, *t*, *r*) with confidence intervals, effect sizes, degrees of freedom and *P* value noted<br>*Give P values as exact values whenever suitable.*
☒	☐	For Bayesian analysis, information on the choice of priors and Markov chain Monte Carlo settings
☒	☐	For hierarchical and complex designs, identification of the appropriate level for tests and full reporting of outcomes
☒	☐	Estimates of effect sizes (e.g. Cohen's *d*, Pearson's *r*), indicating how they were calculated

Our web collection on statistics for biologists contains articles on many of the points above.

Software and code

Policy information about availability of computer code

Data collection	sc-RNA Seq: sc-RNA seq data was performed with the 10X genomics platform. Single cell libraries were generated and sequenced on the Illumina NextSeq500 sequencer.<br>Spatial transcriptomics was performed with the MERFISH imaging was performed on an automated Vizgen Alpha Instrument using imaging buffers, hybridization buffers and parameter files provided by Vizgen.<br>Imaging for IHC was conducted on an Olympus FV3000 confocal microscope using Fluoview software.
Data analysis	sc-RNA Seq: Sequence reads were processed and aligned to the mouse genome using CellRanger 3.0 (10X Genomics). Processing and visualization were conducted with R-package Seurat (https://CRAN.R-project.org/package=Seurat, Satija Lab).<br>MERFISH data was analyzed with custom scripts in R (https://github.com/kaylinker/AgingAstrocytes) and Seurat R-package.<br>All data, unless otherwise stated were plotted with OriginPro 2018 (v 9.6.5)<br>Statistical analysis was conducted with OriginPro 2018 (v 9.6.5)

For manuscripts utilizing custom algorithms or software that are central to the research but not yet described in published literature, software must be made available to editors and reviewers. We strongly encourage code deposition in a community repository (e.g. GitHub). See the Nature Portfolio guidelines for submitting code & software for further information.

Data

Policy information about availability of data

All manuscripts must include a data availability statement. This statement should provide the following information, where applicable:

- Accession codes, unique identifiers, or web links for publicly available datasets
- A description of any restrictions on data availability
- For clinical datasets or third party data, please ensure that the statement adheres to our policy

scRNAseq data sets are available under GEO accession number GSE198027, GSE225741 and GSE226138. MERFISH data sets are available under GEO accession number GSE262083. Human striatal astrocyte aging are available under accession numbers GSE46706 and GSE36192, human striatal astrocyte Huntington's Disease are available under accession number GSE242198, and human Parkinson's Disease are available under accession number GSE157783. Source data for analyses of scRNAseq, MERFISH, immunohistochemistry and RNAscope are provided with this manuscript.

Field-specific reporting

Please select the one below that is the best fit for your research. If you are not sure, read the appropriate sections before making your selection.

[x] Life sciences [ ] Behavioural & social sciences [ ] Ecological, evolutionary & environmental sciences

For a reference copy of the document with all sections, see nature.com/documents/nr-reporting-summary-flat.pdf

Life sciences study design

All studies must disclose on these points even when the disclosure is negative.

Sample size	Sample sizes were selected based on data from the use of similar models by our laboratory and in past studies that are cited in the manuscript.
Data exclusions	No data was excluded from this manuscript
Replication	To verify the reproducibility of the experimental findings, all data collection was done in multiple batches comprising at least four replicates. All experiments were successfully replicated.
Randomization	For all experiments, the mice were randomly allocated to a group.
Blinding	For all analyses, the investigators were blinded to group allocation during data collection, as numerical mouse IDs were the only identifier used.

Reporting for specific materials, systems and methods

We require information from authors about some types of materials, experimental systems and methods used in many studies. Here, indicate whether each material, system or method listed is relevant to your study. If you are not sure if a list item applies to your research, read the appropriate section before selecting a response.

Materials & experimental systems

n/a	Involved in the study	
[ ]	[x]	Antibodies
[x]	[ ]	Eukaryotic cell lines
[x]	[ ]	Palaeontology and archaeology
[ ]	[x]	Animals and other organisms
[x]	[ ]	Human research participants
[x]	[ ]	Clinical data
[x]	[ ]	Dual use research of concern

Methods

n/a	Involved in the study	
[x]	[ ]	ChIP-seq
[x]	[ ]	Flow cytometry
[x]	[ ]	MRI-based neuroimaging

Antibodies

Antibodies used	Primaries:<br>mouse anti-NeuN (Millipore #MAB377)<br>rabbit anti-S100ß (Abcam #ab41548),<br>chicken anti-GFAP (Abcam, #ab4674)<br><br>Secondaries:

	Alexa Fluor 488 goat anti-chicken (A11039)<br>Alexa Fluor 488 goat anti-rabbit(A11008)<br>Alexa Fluor 488 goat anti-mouse (A28175)<br>Alexa Fluor 546 goat anti-mouse (A48255)<br>Alexa Fluor 546 goat anti-rabbit (A28175)<br>Alexa Fluor 647 goat anti-rabbit (A21244)
Validation	The antibodies used in this manuscript have been validated and reproduced by our lab across at least 7 manuscripts by checking cell specificity, background signal, and noting antigen specificity using western blot techniques (Srinivasan et al., 2016; Chai et al., 2017, Nagai et al., 2019; Yu et al., 2020; Diaz-Castro et al., 2019; Endo et al., 2022, Gangwani et al., 2023). All Khakh lab manuscripts.

Animals and other organisms

Policy information about studies involving animals; ARRIVE guidelines recommended for reporting animal research

Laboratory animals	C57BL/6NTac mice were maintained as an in-house breeding colony or purchased from Taconic Biosciences.<br>Both males and females were used in alternating batches.
Wild animals	The study did not involve wild animals
Field-collected samples	This study did not use field-collected samples
Ethics oversight	All experiments were conducted in accordance with the National Institutes of Health (NIH) Guide for the Care and Use of Laboratory Animals and were approved and overseen by the Chancellor's Animal Research Committee (ARC) at the University of California, Los Angeles (UCLA)

Note that full information on the approval of the study protocol must also be provided in the manuscript.
